# Relating glycoprotein structural heterogeneity to function – insights from native mass spectrometry

**DOI:** 10.1016/j.sbi.2019.05.019

**Published:** 2019-07-18

**Authors:** Weston B Struwe, Carol V Robinson

**Affiliations:** Department of Chemistry, Physical and Theoretical Chemistry Laboratory, University of Oxford, South Parks Road, Oxford, OX1 3QZ, UK

## Abstract

Glycosylation is the most complex and prevalent protein modification that influences attributes ranging from cellular localization and signaling to half-life and proteolysis. Glycoconjugates are fundamental for cellular function and alterations in their structure are often observed in pathological states. Most biotherapeutic proteins are glycosylated, which influences drug safety and efficacy. Therefore, the ability to characterize glycoproteins is important in all areas of biomolecular and medicinal research. Here we discuss recent advances in native mass spectrometry that have significantly improved our ability to characterize heterogeneous glycoproteins and to relate glycan structure to protein function.

## Introduction

**Figure 1:**
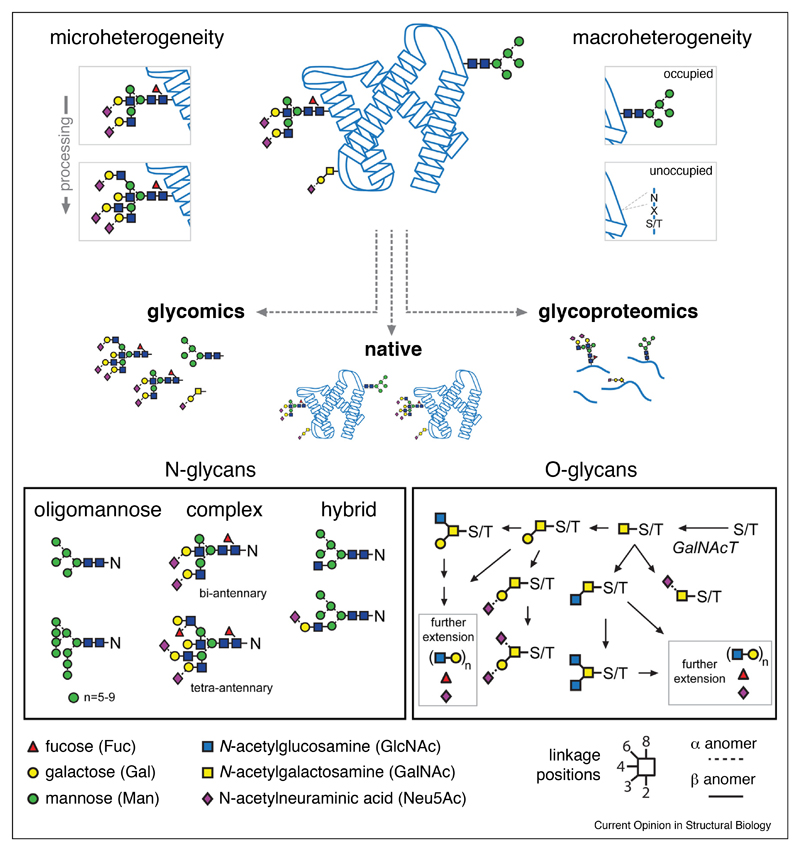
Mass spectrometry strategies for glycoproteins focus on structural analysis of isolated glycans (glycomics), compositional and positional glycosylation from enzyme digested peptides (glycoproteomics) and global evaluation of intact glycoprotein assemblies by native analysis. Glycoprotein heterogeneity arrises from variable glycan structures (microheterogeneity) and different degrees of occupancy at a given site (macroheterogeneity). *N*-Glycans are covalently attached to asparagine residues containing an N-X-S/T sequon and are classified as oligomannose, complex or hybrid type. *O*-Glycans do not have specific amino acid sequence requirements and can be linked to serine or threonine residues. Both *N*-glycans and *O*-glycans are synthesized in a non-template driven manner producing a range of structures with different monosaccharide compositions, linkages and topologies.

The modification of proteins by complex oligosaccharides, commonly referred to as glycans, is highly diverse and influences overall structure and function. Glycosylation is the most prevalent protein post-translational modification (PTM) in eukaryotes, with up to 50% of the human proteome being covalently modified by sugars [1,2]. Glycans are central to cellular function, and changes in glycosylation are observed in many diseases, including different cancers [3], diabetes [4], neurodegeneration [5], and aging [6]. The importance of glycosylation is exemplified by the human disease Congenital Disorders of Glycosylation where mild loss-of-function in glycosylation machinery results in severe multisystem dysfunction, organ failure, and premature death [7]. Glycans are complex structures that differ between cell types and are temporally regulated. The role of glycans on a given protein is specific, and changes in their structures can fine-tune protein function. Adding to this complexity is the extent to which a protein can be modified (termed macroheterogeneity) and the range of glycan structures that exist at a single glycosylation site (microheterogeneity), making structural studies challenging (Figure 1).

Glycosylation occurs through different mechanisms and locations within the cell. The current understanding of glycan biosynthesis helps glycobiologists assign functional roles of glycans to individual proteins. The most well-known and frequent type is *N*-linked glycosylation, where a preassembled glycan donor is covalently attached to asparagine residues, specifically Asp-X-Ser/Thr sequons, on nascent polypeptides in the lumen of the endoplasmic reticulum (ER) [8,9]. *N*-Glycans are then processed in the Golgi apparatus by glycosidase and glycosyltransferase enzymes to form high-mannose, hybrid and complex structures (Figure 1). *O*-Glycosylation also occurs in the Golgi where *N*-acetylgalactosamine (GalNAc) residues are added to Ser/Thr residues and extended by step-wise addition of galactose (Gal), *N*-acetylglucosamine (GlcNAc), fucose (Fuc) and *N*-acetylneuraminic acid (Neu5Ac) [10]. Monosaccharides are connected by a range of linkages creating diverse topologies and carbohydrate epitopes, such as the blood group antigens observed on red blood cells.

Another important type of glycosylation is the reversible addition of single GlcNAc residues at Ser/Thr amino acid residues by *O*-GlcNAc transferase (OGT), and subsequent removal by *O*-GlcNAcase. These processes are believed to interconnect in cell signaling mediated by protein phosphorylation [11]. In the last decade, glycomics has grown considerably, but the field has trailed proteomics and genomics in the development of analytical tools to dissect the inherent structural complexity of glycoproteins. However, very recent advances in native mass spectrometry (MS), namely the development of high-resolution instruments capable of transmitting large protein ions, has transformed the characterization of intact glycoproteins and their interactions, thus providing a new way to investigate functional roles of sugars.

Native MS is a principal tool for structural biology, yielding low-resolution structural data and measurements of the stoichiometry of macromolecular complexes, as well as the kinetics of complex assembly, the dynamics of assembled complexes and characteristics of ligand binding [12–15]. Native MS is also complementary to traditional structural methods, such as X-ray crystallography and cryoEM, since it provides a means to identify protein assemblies with relatively minimal sample requirements. X-ray crystallography and cryoEM are not amenable for glycoproteins due to the inherent flexibility and heterogeneity of oligosaccharides. This has been referred to as the ‘glycosylation problem’ [16,17], which is typically circumvented by chemical or enzymatic modulation to remove or simplify protein glycosylation. Although valuable, these structures result in non-wild type proteins without the full repertoire of PTMs. Glycomics and glycoproteomics analyses help fill these gaps by providing information on glycan structure (i.e. microheterogeneity) and location on the peptide backbone (i.e. macroheterogeneity), respectively.

Similarly, native MS does not replace glycomics or glycoproteomics investigations, but provides additional structural and biophysical information, arguably not achievable by conventional methods. Despite the relative newness of native MS, its potential is noteworthy, evident from various investigations that tackle heterogeneous glycoprotein assemblies. In this review, we discuss the latest reports demonstrating the utility of native MS to characterize glycosylation and link structural features to protein function.

## Biotherapeutic glycoproteins

The majority of biotherapeutic proteins are *N*-glycosylated and include monoclonal antibodies (mAbs), hormones, growth factors and vaccines. MAbs not only lead the biopharmaceutical market ($75 billion in 2014), but are also projected to grow to $120 billion by 2020, especially with ‘biosimilars’, next-generation drugs developed from innovator products, and the emergence of engineered fusion proteins, or chimerics, for enhanced functionality and stability [18,19]. Glycosylation can significantly alter mAb safety and efficacy. Therefore, understanding glycan structure and occupancy is critically important to comply with drug regulatory policies. MS of intact biotherapeutics is gaining acceptance, and commercial availability of mass spectrometers capable of measuring protein assemblies has accelerated its use. A pivotal stride in glycoprotein analysis was made by applying a high-resolution Orbitrap-based instrument to a range of soluble proteins, including an immunoglobulin gamma (IgG) mAb [20^•^]. IgG glycoforms were resolved, revealing 162 Da mass shifts corresponding to hexose (presumably galactose) monosaccharides. Although IgGs are only ~2% carbohydrate by mass, this suggested MS can detect glycan heterogeneity on intact glycoprotein assemblies without prior liquid chromatography (LC) separation or sample modification.

**Figure 2:**
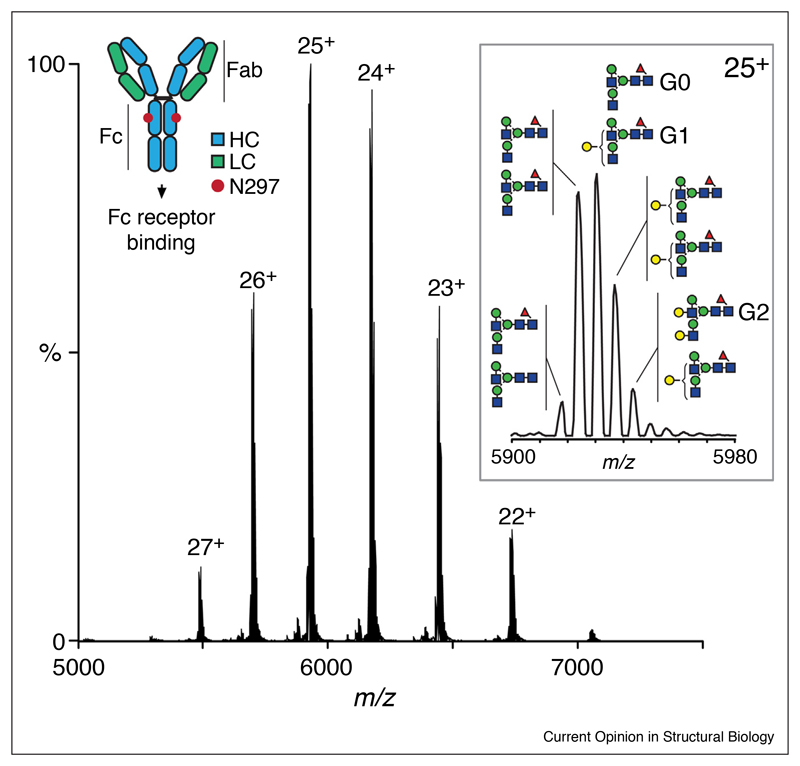
Native mass spectrum of monoclonal IgGs (150 kDa) display a characteristic charge-state distribution of 21–27+ centering at 6000 *m/z* (trastuzumab (Herceptin) is shown). The pairing of the two conserved complex-type *N*-glycans, commonly referred to as GO, G1, and G2, present on each heavy chain (HC) in the Fc domain (Asn 297) are well-defined. Native MS can easily identify glycan structural features such as core fucosylation (red triangles) and terminal galactosylation (yellow circles), which are important attributes in biotherapeutic activity.

Many native glycoprotein studies to date have focused on therapeutic mAbs due in large part to application in industry, the wealth of glycan information provided by MS and the fact that they provide a relatively straightforward system for study [21–24]. IgGs have two comparatively simple biantennary *N*-glycans, with variability in terminal galactosylation/sialylation and core-fucosylation. IgG fucosylation is an important feature that affects receptor binding affinity and antibody-dependent cellular cytotoxicity (ADCC), the desired effect in cancer therapy. IgGs are 150 kDa complexes composed of two heavy and two light chains that characteristically have charge state distributions between 22 and 28+ when measured under non-denaturing conditions (Figure 2). Since IgGs have two glycans in the Fc domain, the pairing of structures (commonly termed GO, G1, and G2, corresponding to the number of galactose residues) can be readily measured. Native MS offers a rapid screening technique for favorable or harmful glycosylation that may arise during mAb bioprocessing [25] and has also been used to quantify drug to antibody ratios in the preparation of mAb drug conjugates (ADCs) [24,26–28].

In addition to glycosylation, native MS can simultaneously screen for other important PTMs that may can occur during mAb production, including C-terminal lysine clipping, disulphide bonding, N-terminal glutamine cyclization, methionine oxidation and deamidation of asparagine residues. To accurately assign PTMs, prior knowledge of the underlying amino acid sequence is essential in order to relate the observed experimental mass to the predicted mass. Mass measurements under non-denaturing conditions are beneficial, as compared to top–down mass spectrometry, due to the improved peak separation arising from the lower charge state distribution. Importantly, native MS can also be used to assess antigen binding and interactions with Fc gamma receptors, for which glycosylation can affect IgG affinities.

Native MS can also be used to examine IgG stability arising from subtle variations in glycosylation [29,30], domain exchange [31], biosimilarity [32], as well as disulphide bonding among IgG subclasses [29]. These experiments are based on collision-induced unfolding (CIU), where ion mobility (IM) is used to monitor gas-phase unfolding of proteins subjected to increasing kinetic energies by collision induced dissociation (CID). A recent study of truncated IgG glycoforms, generated by sequential exoglycosidase digestion, revealed how Fc glycosylation influences stability in the gas phase. Interestingly, the absence of IgG *N*-glycans, which reside in the interstitial space between both CH2 domains, resulted in the greatest loss in stability. A subsequent experiment demonstrated stability using IgGs treated with endoglycosidase S, an immune evasion factor secreted by *Streptococcus pyogenes* that disrupts IgG-mediated effector functions by glycan trimming [33]. These results are notable in that they demonstrate how MS reveals the contributions of glycans to the overall rigidity of glycoprotein assemblies.

Other than mAbs, native MS studies of therapeutic glycoproteins are limited in number, but existing publication are nonetheless important in that they substantiate native MS in quality control studies. For example, recombinant erythropoietin (EPO) is routinely used to treat anaemia by stimulating erythrocyte production. EPO is a heavily glycosylated hormone (~40% carbohydrate by mass) with one *O*-glycan and three conserved *N*-glycans present as bi-antennary, tri-antennary and tetra-antennary structures. Pharmacokinetic studies of EPO have shown that glycan sialylation and the degree of branching improves *in vivo* serum half-life and activity [34,35]. A comparative analysis, combining glycoproteomics and native MS, scored biosimilarity between several recombinant human EPO (rhEPO) formulations and showed that sialylation across *N*-glycans and *O*-glycans could be quantified [36]. Interestingly, non-human *N*-glycolylneuraminic acid (Neu5Gc) residues were detected, which are known to occur on 1% of EPO expressed in Chinese hamster ovary (CHO) cells [37], the industrial standard for biotherapeutic production. Neu5Gc is immunogenic and targeted by circulating anti-Neu5Gc antibodies in humans [38]. To ensure safety, Neu5Gc content is required to be within a certain range on biopharmaceuticals. This study demonstrates the strength of native MS for characterizing critical quality attributes on glycosylated proteins that can be demanding by existing methods. For instance, sialic acid speciation by high-performance LC (HPLC) requires 50–100 μg of glycoprotein, compared with ~1 μg for a single MS analysis.

Native MS of next generation biologics and vaccines will prove challenging because of their heterogeneity and will test the boundaries of high-resolution MS instruments. Such a challenge is exemplified by the anti-inflammatory drug etanercept, a dimeric fusion protein containing an anti-tumor necrosis factor and an Fc domain linked by an extensively *O*-glycosylated peptide linker. The mass of this protein has been routinely reported as 150 kDa with *N*-glycans and *O*-glycans contributing 50 kDa to this mass [39,40]. Only recently has this value been refined to 130 kDa from a native MS analysis using exoglycosidase digestions [41^•^]. Etanercept has 6 *N*-glycans and 26 potential *O*-glycosylation sites, and the intact spectrum was deemed too complex to assign all the glycoforms, which were narrowly resolved following zero-charge state deconvolution. Subsequent sialidase digestion simplified the spectra considerably, and after complete *N*-glycan removal by peptide *N*-glycosidase F (PNGase F), the number of *O*-glycans present on etanercept could be assigned (from 14 to 23 of the 26 putative sites).

Reporting glycosylation, specifically the presence or absence of glycans on IgG subunits, is required by the European Medicines Agency [42], and native MS is a prospective method to rapidly characterize these features. Alternative methods for mass analysis of intact glycoproteins by orthogonal methods can be complicated or inaccurate. For instance, glycans influence the electrophoretic behavior of proteins when performing SDS-PAGE [43]. Biotherapeutic glycoproteins will undoubtedly continue to propel the field; however, the full potential of native MS, specifically for studying protein assemblies and interactions, remains to be explored.

## Towards glycan-specific interactions

The ability to measure assembly, kinetics and substrate interactions of noncovalent macromolecular complexes is one of the great strengths of native MS. Arguably, this aspect has the greatest potential for glycoproteins, particularly high-resolution analysis of glycoprotein subunits and their influence on ligand binding. We expect that these studies will emerge in a fashion similar to earlier investigations of small molecule binding, exploiting high-resolution instrumentation [44,45]. There are several studies in which the interactions and oligomerization between glycoproteins were determined by lower-resolution time-of-flight (ToF) mass analyzers. One excellent example describes the initial stages of the Complement pathway showing that hexamers of IgGs assemble *via* noncovalent interactions of Fc domains [46]. Three gain-of-function mutations allowed assembly of 150 kDa IgG subunits into 890 kDa hexamers. This was followed by an MS study that identified additional amino acids involved in hexamer formation [47]. Importantly, this novel hexa-body approach potentiated the cytotoxic effect of a therapeutic antibody.

More recently, the benefits of high-resolution Orbitrap mass analysers became evident with the identification of previously unreported *N*-glycosylation and *O*-glycosylation sites on human C9 (discussed below) and C8 proteins, involved in the complement pathway [48^••^,^49^]. The C8 complex consists of α, β and γ subunits, of which α and β are *N*-glycosylated, and Trp residues can be C-mannosylated. The intact MS of C8 showed two distributions, one matching the C8αβγ complex and a second C8αβ complex that was postulated to arise during sample preparation. Approximately twenty peaks were detected for the most abundant C8αβγ glycoform, having two *N*-glycans (di-sialylated biantennary) with seven C-mannose residues. Other interesting modifications included *O*-glycans on a Tn antigen (i.e. single *O*-linked GalNAc) and Man5GlcNAc2 (Man5) and Man8GlcNAc2 (Man8) oligomannose-type *N*-glycans. This report was notable for the discovery of *O*-glycans on C8γ subunits. In addition, the quantitative and qualitative coverage across all subunits of three different glycan classes will certainly provide insight into their roles in assembly and biological function. Similar to the evaluation of EPO discussed above, the analysis of C8 benefited greatly from a hybrid approach incorporating glycoproteomics. These results demonstrate the benefit of native MS and how it can guide structural studies by revealing new glycosylation patterns, which may be overlooked by conventional methods.

Recently, high-resolution MS analysis has been used to examine the effects of carbohydrate modification on drug-protein and protein–protein interactions. For example, alpha-1-acid glycoprotein (AGP) is a serum protein with five complex type *N*-glycans [50^••^]. The commonly used anticoagulant drug warfarin bound weaker to AGP glycoforms with increased *N*-glycan branching and terminal fucosylation. Surprisingly, the dissociation constant of warfarin binding varied for sixteen individual glycoforms of AGP. Furthermore, the interaction between haptoglobin (Hp), a glycoprotein with eight *N*-glycans, and haemoglobin (Hb) heterodimers were glycan-specific. *N*-Glycan branching reduced Hp–Hb affinity while outer-arm fucosylation on Hp stabilized these interactions. Most recently, glycan binding specificity between two lectins *Aleuria aurantia* lectin (AAL) and *Phaseolus vulgaris* leucoagglutinin (PHA-L) to AGP and Hb were described [51]. This lectin-purification native MS approach has considerable potential not only for identifying *N*-glycan structural details, including terminal fucosylation and the degree of branching, but also for understanding multivalent interactions, which are characteristic features of glycan binding proteins. Overall, these results demonstrate how native MS can reveal individual glycan structure–function relationships within glycoprotein oligomers, lectin complexes, and protein–glycoprotein complexes, not achievable by other biophysical techniques.

## The role of the biosynthetic pathway

Glycan assembly is an intricate process, taking place in different locations within eukaryotic cells. Some actions are conserved, such as initial lipid-linked oligosaccharide (LLO) assembly and protein attachment in the ER, but subsequent structural processing in the Golgi deviates considerably across species [52]. Because of the presence of isomeric monosaccharides on a given glycoprotein (e.g. Man and Gal = 162.1 Da; GalNac and GlcNAc = 203.1 Da), the initial information from a native MS spectrum only provides compositional data for glycans. Further interpretation can also be facilitated by existing glycomics and glycoproteomics datasets, such as reported glycan structures (i.e. compositional information) and the degree of glycan occupancy on a given glycoprotein. In addition to existing glycomics data, knowledge of cellular glycosylation also provides guidelines for MS interpretation [53], such as the cell line for protein expression (e.g. CHO versus human embryonic kidney (HEK) cells) for which the sialic acid linkages are well-defined [54]. However, making assumptions regarding glycan biosynthetic ‘rules’ precludes the discovery of unusual glycoforms or glycosylation occupancy.

The analysis of the complement C9 protein is a good example of native MS challenging these ‘rules’. Three *N*-glycans were identified despite the presence of only two requisite Asp-X-Ser/Thr sequons [48^••^]. The third *N*-glycan, albeit lowly expressed, was located at an Asp-Ala-Ala amino acid sequence that was confirmed by glycoproteomics. Similar to C8, *O*-glycans, which have been predicted on C9, were identified as Hex-HexNAc disaccharides (likely core-1) plus one or two sialic acids. The C9 protein is part of the complement membrane attack complex that enters the outer membrane to self-assemble into asymmetric barrels that rupture target cells. Interestingly, the location of *N*-glycans in the transmembrane region, implicates these glycans in critical roles of function or assembly.

**Figure 3:**
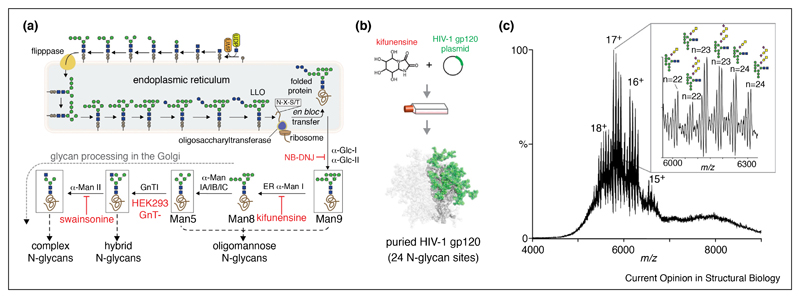
(**a**) Glycoengineering strategies direct *N*-glycan biosynthetic enzymes in the endoplasmic reticulum (ER) and early Golgi to generate homogeneous glycoforms. Small molecule inhibitors such as NB-DNJ, kifunensine, and swainsonine, as well as the HEK293 GnT-I knock-out cell line, are particularly useful for delineating glycan composition. (**b**) For example, kifunensine, which blocks processing to produce Man9GlcNAc2 *N*-glycans, was recently used to assign glycosylation occupancy of recombinant HIV-1 gp120 (adopted from PDB: 5ACO). (**c**) Global *N*-glycan occupancy of gp120 could be quantified simply by evaluating the number of Man9GlcNAc2 moieties (inset). Additionally, simplification of protein *N*-glycosylation revealed the presence and number of *O*-glycans. Figure adapted from Ref. [45].

The current understanding of glycan biosynthesis is helpful in that it may be manipulated chemically or enzymatically to generate informative glycoforms for intact MS. One example used the potent ER mannosidase inhibitor kifunensine to quantify glycosylation occupancy on HIV-1 gp120 [55^••^]. Kifunensine blocks glycan processing resulting in Man9GlcNAc2 (Man9) at each occupied *N*-glycan site, and the ensuing MS analysis of kifunensine-treated glycoproteins made determination of quantitative *N*-glycan occupancy straightforward and accurate (Figure 3). In this way, only the number and relative abundance of Man9 glycans needed to be determined to assign global glycoprotein occupancy. An HIV-1 gp120 strain, with 24 potential *N*-glycosylation sites, was also examined to identify how many sites were glycosylated or occupied. Results showed that gp120 glycoforms were 40.6% occupied (i.e. all 24 sites contained *N*-glycans), followed by 41.6% (*n* = 23, gp120 with one unoccupied *N*-glycan site) and 17.8% (*n* = 22, gp120 with two unoccupied *N*-glycan sites). Furthermore, the intact analysis identified the presence of *O*-glycosylation on gp120 similar to the aforementioned C9 and C8 studies. These results are important for HIV vaccine development as many broadly neutralizing antibodies, which can be elicited by recombinant mimics of the HIV envelope glycoprotein, interact with exposed gp120 glycans. Notably, this approach is applicable to any recombinant glycoprotein and is more accurate for quantitative glycan occupancy assessment compared to existing peptide-based methods because native MS measures all glycoforms simultaneously.

Glycoengineering is beneficial for structural studies and will become critically important in native MS both for quantitative purposes, as shown with gp120, but also in solving looming ‘glycosylation problems’ [16,17]. Restricting glycan heterogeneity is a common methodology in structural biology, and there are several glycosylation enzyme inhibitors and cell lines available for this purpose (Figure 3). For example, HEK293 GnTI-cells, which produce Man5 glycans, are commonly used for generating relatively simple glycoforms for X-ray crystallography [56]. Similar to the use of exoglycosidases discussed above, the ability to manipulate glycan biosynthesis during recombinant protein production will become useful in resolving intact glycoproteins and associated complexes by MS.

## Outlook

The power of an integrated approach for analyzing glycoproteins is evidenced by native MS supporting existing methods and providing numerous forms of structural information not achievable by any other single method. Although informative, high-resolution MS analysis of intact glycoproteins is limited in that it does not capture the structural complexity of individual glycans or their positional information on proteins. Nevertheless, the advantages of native MS include monosaccharide compositional analysis, quantitative glycan occupancy, glycoprotein–protein/drug interactions and the identification of new glycoforms. Unquestionably, native MS holds tremendous potential in both industrial and basic research, and we expect that its use will continue to expand. The success of glycomics, glycoproteomics, as well as the use of native MS for global evaluation of intact glycoprotein assemblies, will be encouraged by the development and availability of protocols, dissemination of data, development of repositories and the creation of software for their analysis. One principal goal among structural biologists, is linking protein function to glycan structure. Native MS is arguably a leading, yet emerging, method to explore these complex relationships. We anticipate considerable progress in the coming years enabling new insight on long-standing questions in structural glycobiology.
